# Physician and patient attitudes towards complementary and alternative medicine in obstetrics and gynecology

**DOI:** 10.1186/1472-6882-8-35

**Published:** 2008-06-26

**Authors:** Mandi L Furlow, Divya A Patel, Ananda Sen, J Rebecca Liu

**Affiliations:** 1 Department of Obstetrics and Gynecology, Harvard University, Boston, Massachusetts, USA; 2 Department of Obstetrics and Gynecology, University of Michigan, Ann Arbor, Michigan, USA; 3 Center for Statistical Consultation and Research, University of Michigan, Ann Arbor, Michigan, USA

## Abstract

**Background:**

In the U.S., complementary and alternative medicine (CAM) use is most prevalent among reproductive age, educated women. We sought to determine general attitudes and approaches to CAM among obstetric and gynecology patients and physicians.

**Methods:**

Obstetrician-gynecologist members of the American Medical Association in the state of Michigan and obstetric-gynecology patients at the University of Michigan were surveyed. Physician and patient attitudes and practices regarding CAM were characterized.

**Results:**

Surveys were obtained from 401 physicians and 483 patients. Physicians appeared to have a more positive attitude towards CAM as compared to patients, and most reported routinely endorsing, providing or referring patients for at least one CAM modality. The most commonly used CAM interventions by patients were divergent from those rated highest among physicians, and most patients did not consult with a health care provider prior to starting CAM.

**Conclusion:**

Although obstetrics/gynecology physicians and patients have a  positive attitude towards CAM, physician and patients' view of  the most effective CAM therapies were incongruent. Obstetrician/gynecologists should routinely ask their patients about their use of CAM with the goal of providing responsible, evidence-based advice to optimize patient care.

## Background

Complementary and Alternative Medicine (CAM) is defined by the U.S. National Center for Complementary and Alternative Medicine (NCCAM) as a group of diverse medical and healthcare systems, practices, and products that are not presently considered to be part of conventional medicine [1]. A landmark study by Eisenberg et al. published in 1993 was the first national survey of the use of CAM by the adult American public. This study estimated that one in three adults (34%) had used at least one complementary therapy during the past year and that this population made an estimated 425 million visits to practitioners of complementary therapy [2]. A follow-up national survey documented a 25% increase in prevalence of CAM use between 1990–1997 [3]. Between 1997 and 2002, these trends remained stable, and CAM use was reported by 72 million U.S. adults.

Although the use of CAM to supplement conventional medical treatment is common among patients, attitudes and use of CAM among physicians is more controversial. A study by Jump et. al. demonstrated that the majority of physicians located in a southeastern city in the United States still viewed the majority of CAM therapies as not part of legitimate medical practice [4]. In addition, Milden et. al. found that while a random sample of California physicians demonstrated an overall positive attitude toward CAM, 61% still found themselves discouraging CAM therapies because they are not knowledgeable enough about the safety or efficacy of CAM treatments. The majority (80%) of physicians preferred to rely exclusively on conventional biomedical treatments [5]. Similarly, at the Mayo Clinic in Rochester, MN, a survey of internal medicine physicians revealed that although most physicians agreed that some CAM therapies hold promise for the treatment of symptoms or diseases, most physicians were not comfortable in counseling patients about CAM treatments [6]. In contrast, meta-analysis of the survey literature as well as several individual national surveys indicate that there is significant interest in CAM among physicians from varying subspecialties [7-10].

The high prevalence of CAM use among adults in the United States suggests that there is a positive attitude towards CAM use among this population [11]. Disparate attitudes and use of CAM among physicians and patients could result in limited disclosure of the patient's use of alternative therapies to their physician. In a study by Eisenberg et. al., 72% of the patients used alternative medicine without informing their physicians [3]. This could lead to significant risks to the patient including delay or avoidance in obtaining the appropriate conventional treatment, incorrect diagnosis, interference with the mechanism of action of a prescribed medication, or harmful reactions from ingested substances [12]. On the other hand, while the study by Jump et. al. found that the most physicians feel that CAM modalities are not part of legitimate medical practice, nearly two-thirds of these same physicians had prescribed or referred patients for at least one complementary therapy [4]. Furthermore, CAM is becoming more mainstream within the healthcare system as demonstrated by the integration into medical school curriculum, reimbursement by some third-party payers for selected alternative therapies, and the development of the U.S. National Center for Complementary and Alternative Medicine (NCCAM) at the National Institutes of Health [3,12-15]. In fact, NCCAM established a CAM education project in 2000, with the goal of incorporating CAM information into medical, dental, nursing, and allied health professions schools' curricula, into residency training programs, and into continuing education courses [16,17]. In 2004, curriculum guidelines in integrative medicine for medical schools were published by the Education Working Group of the Consortium of Academic Health Centers for Integrative Medicine (CAHCIM) [18]. To date, thirty-nine medical schools in North America currently belong to CAHCIM, all of whom offer medical education, research, and/or clinical services in integrative medicine [19]. The ability of physicians to inquire and educate about CAM modalities is becoming increasingly important.

With one of the largest subgroups of CAM users being reproductive age, educated, employed women [20], the obstetrician gynecologist plays an integral role in incorporating CAM use with conventional medicine. In 1999, The American College of Obstetricians and Gynecologists (ACOG) published a Committee Opinion on the role of CAM in clinical practice encouraging its members to counsel their patients about their motivation for and use of CAM and to provide information on its safety and effectiveness [21]. The goal of the current study was to examine the attitudes toward and use of CAM specifically among obstetrics and gynecology patients and physicians.

## Methods

The surveys used in this study were reviewed by the Institutional Review Board of the University of Michigan Medical School (IRBMED). The study was exempted from IRBMED review as completion of the surveys were considered consent to participate. Furthermore, no direct identifiers were included on the surveys.

### Physician survey

All practicing obstetrician/gynecologists in the state of Michigan who were members of the American Medical Association in 2004–2005 were included in the sample (n = 1009). A packet containing a cover letter and the survey was mailed to all physicians in the sample. The survey instrument ascertained information regarding the physician's view of the effectiveness of 17 different CAM modalities, use of each CAM modality within their medical practice, and general attitudes and beliefs toward CAM. Demographic information, including age, gender, type of medical degree, year of medical school graduation, specialty, and ethnicity, was ascertained. No direct identifiers were included on the surveys, and return of the survey was considered consent to participate.

As the initial response rate was below 23% (n = 231) and those who returned surveys could not be identified, the survey was mailed to the entire sample a second time. Physicians were requested to return surveys only if they had not responded to the first request. The response rate after the second mailing was 41.0% (n = 401). Twenty (5.0%) surveys were excluded from the statistical analyses due to substantially incomplete data.

### Patient survey

A convenience sample of all women who presented to the University of Michigan Taubman Health Care Center during May 2005 for an obstetric/gynecologic visit comprised the patient sample in this study. Patients were given a questionnaire with their check-in paperwork, and completion of the survey was considered consent to participate. During the survey collection period, 1519 patients were seen, and 483 women completed questionnaires, resulting in a response rate of 32%. Three surveys (0.6%) were excluded from the statistical analyses due to substantially incomplete data. The survey ascertained information regarding patients use of one or more CAM modalities *specifically *for the treatment of obstetric or gynecologic problems, including menstrual or menopausal symptoms, pelvic pain, libido, infertility, contraception, pregnancy symptoms, or labor induction or augmentation. Further information regarding how the patient learned about CAM, average monthly expenditures on CAM, general attitudes toward CAM, and income level was collected. No personal identifying information was included on the surveys.

### Statistical Analysis

Attitudes and practices of physicians and patients regarding CAM were characterized. On the physician survey, three sets of questions (i.e., view of effectiveness of CAM modalities, use of CAM approaches in practice, and general attitudes towards CAM) were categorized for purposes of statistical analysis. With respect to effectiveness, responses were categorized as highly/moderately, seldom/not at all/neutral, or harmful. Regarding use of CAM approaches in practice, responses were categorized as endorse/provide/refer or would not recommend. For general attitudes, responses were categorized as agree, disagree, or neutral/skipped. Multivariable logistic regression analyses were conducted to examine associations of physician age, gender, and race with (1) the belief that CAM approaches hold promise for the treatment of symptoms, conditions, and/or diseases; and (2) the belief that CAM approaches have no true impact on treatment of symptoms, conditions, and/or diseases.

Patients were asked if they had ever used specific CAM modalities for a variety of obstetric and/or gynecologic problems (i.e., menstrual or menopausal symptoms, pelvic pain, libido, infertility, contraception, pregnancy symptoms, and labor induction or augmentation). The respondent was considered to have used a specific CAM modality if she indicated ever using it for any of the obstetric and/or gynecologic problems queried; the respondent was considered to have never used a specific CAM modality if she indicated "have not used." Five items under general attitudes were positively framed with the remaining two framed negatively. The latter were reverse coded before calculating the attitude block-score so that an overall lower score would be indicative of a positive attitude. Additionally, a dichotomous measure was created from the responses of each of the seven items under *general attitudes *scale that is coded as 1 for responses "strongly agree" or "agree" and as 0 otherwise. These dichotomous variables thus can be envisioned as indicator of agreement to the item statement. General attitudes of physicians and patients towards CAM were compared by means of two sample chi-square tests of proportion applied to each item. All statistical analyses were conducted using SPSS version 15 (SPSS Inc., 2006, Chicago, IL) for Windows and SAS version 9.1 statistical software (SAS Institute, Inc., Cary, NC).

## Results

### Characteristics of the Study Population

#### Physicians

We received 401 surveys from physicians, for a final response rate of 41%. Of these 401 surveys, 396 (98.8%) had complete information and were included in the analyses. Over half (57%) of physician respondents were male; 41% were female and 2% did not report gender. Most physicians self-identified as Caucasian (81.4%), and the rest as African American (4.2%), Asian (7.1%), Hispanic (1.3%), multi-racial (0.8%), or other (1.3%). Median age of physicians was 48 years (range: 30–83) and the median year of graduation from medical school was 1984 (range: 1945–2002). Most physicians had attained an M.D. degree (95.8%), and the remaining respondents either had a D.O. degree (1.8%) or did not report their degree (2.4%).

#### Patients

We received 483 surveys from patients who were seen at the University of Michigan outpatient obstetrics and gynecology clinic during the study period, with a final response rate of 32%. 480 patient surveys with complete information were included in the analyses.

### General Attitudes towards CAM

Surprisingly, physicians appeared to have a more positive attitude towards CAM as compared to general obstetric/gynecology patients (Table 1). Most physicians indicated that clinical care should integrate the best conventional and CAM practices (73.8%), whereas only 40.8% of patients agreed with this statement (p < .05, 95% confidence interval [0.27, 0.39]). Similarly, more than half of the physicians respondents indicated that CAM includes areas and methods from which conventional medicine could benefit (73.2%), that CAM approaches hold promise for treatment of symptoms, conditions and diseases (59.3%), that health professionals should be able to advise their patients about commonly used CAM methods (68%), and that knowledge about CAM is important to them as patients (54.9%). Less than 50% of patients agreed with each of these statements (p < .05 for all statements). Although both physicians and patients disagreed with the statements: while a few CAM approaches may have limited health benefits, they have no true impact on treatment of symptoms, conditions and/or diseases, or CAM is a threat to public health, a higher proportion of physicians disagreed with these statements (p < .05, confidence intervals [0.13, 0.26] and [0.17, 0.30] respectively).

**Table 1:** Physician and patient attitudes towards complementary and alternative medicine (CAM)

**Statement**	**Agree (%)**	**Disagree (%)**	**Neutral/Skipped (%)**
Clinical care should integrate the best conventional and CAM practices.*			
Physician	281 (73.8)	29 (7.6)	71 (18.6)
Patient	196 (40.8)	4 (0.8)	280 (58.3)
CAM includes areas and methods from which conventional medicine could benefit.*			
Physician	279 (73.2)	23 (6.0)	79 (20.7)
Patient	192 (40.0)	10 (2.1)	278 (57.9)
CAM approaches hold promise for treatment of symptoms, conditions and/or diseases.*			
Physician	226 (59.3)	39 (10.2)	116 (30.5)
Patient	169 (35.2)	12 (2.5)	299 (62.3)
While a few CAM approaches may have limited health benefits, they have no true impact on treatment of symptoms, conditions and/or diseases.*			
Physician	93 (24.4)	178 (46.7)	110 (28.9)
Patient	51 (10.6)	129 (26.9)	300 (62.5)
Health professionals should be able to advise their patients about commonly used CAM methods.*			
Physician	259 (68.0)	26 (6.8)	96 (25.2)
Patient	209 (43.5)	19 (4.0)	252 (52.5)
CAM is a threat to public health.*			
Physician	34 (8.9)	252 (66.1)	95 (24.9)
Patient	23 (4.8)	205 (42.7)	252 (52.5)
Knowledge about CAM is important to me as a patient.*			
Physician	209 (54.9)	56 (14.7)	116 (30.5)
Patient	171 (35.6)	20 (4.2)	289 (60.2)

* P-value is for comparison of strongly agree/agree vs. disagree/neutral/skipped; all p values < .001.

Among physicians, female physicians were 5.9 times more likely  (95% CI: 1.7–21.3) to believe that CAM approaches hold promise for the treatment of symptoms, conditions and/or diseases, after adjusting for age and race. Increasing physician age, after adjusting for race and gender, was significantly associated with the belief that CAM approaches have no true impact on treatment of symptoms, conditions, and/or diseases (OR = 1.03; 95% CI: 1.01–1.06).

### Physician Attitudes Regarding Effectiveness of Specific CAM Modalities

Overall, most physicians had a positive attitude regarding the effectiveness of specific CAM modalities. As shown in Table 2, CAM modalities most frequently cited by physicians as being highly or moderately effective included biofeedback (73.8%), chiropractic (65.6%), acupuncture (62.4%), and meditation (61.9%). Few physicians viewed any of the CAM modalities queried as harmful to patients.

**Table 2:** Physician opinion of effectiveness of CAM modalities.

**CAM Modality**	**Highly/Moderately Effective (%)**
Biofeedback	73.8
Chiropractic	65.6
Acupuncture	62.4
Meditation	61.9
Hypnosis/Guided imagery	46.2
Herbal medicine	41.2
Music therapy	36.8
Therapeutic touch	36.8
Traditional Chinese medicine	27.8
Homeopathy	24.9
Special diets	23.4
Bioelectromagnetic therapies	20.2
Aromatherapy	13.6

### Physician Approaches to CAM in Practice

Most (97.6%) physicians surveyed routinely endorsed, provided or referred patients for treatment utilizing at least one CAM modality. Many did so for a wide variety of CAM modalities. As shown in Table 3, the most commonly reported CAM modalities endorsed, provided or referred by physicians included movement therapies (86.4%), biofeedback (80.3%), acupuncture (79.8%), meditation (78.0%), chiropractic (70.9%), and hypnosis/guided imagery (70.1%). On the other hand, over half of physicians would not recommend the use of homeopathy, bioelectromagnetic therapies, or aromatherapy. In addition, a sizeable proportion of physicians would not recommend special diets (47.8%) or traditional Chinese medicine (47.2%). While the greatest proportion of physicians viewed herbal medicine and homeopathy as harmful, over 40% of physicians reported that they endorse, provide or refer their patients for those modalities.

**Table 3:** Physician approach to CAM in practice

**CAM Modality**	**Endorse/Provide/Refer (%)**	**Would Not Recommend (%)**	**Missing (%)**
Movement therapies	86.4	10.5	3.1
Biofeedback	80.3	12.1	7.6
Acupuncture	79.8	18.1	2.1
Meditation	78.0	17.6	4.4
Chiropractic	70.9	25.5	3.6
Hypnosis/Guided imagery	70.1	25.2	4.7
Herbal medicine	61.4	33.4	5.2
Music therapy	59.3	34.7	6.0
Therapeutic touch	56.7	37.5	5.8
Special diets	45.9	47.8	6.3
Traditional Chinese medicine	43.3	47.2	9.5
Homeopathy	41.7	52.0	6.3
Bioelectromagnetic therapies	40.4	52.5	7.1
Aromatherapy	32.3	61.7	6.0

### Patient Use of CAM

Overall, 262 (54.5%) patients reported ever using at least one type of CAM modality for obstetric and/or gynecologic problems. As shown in Table 4, the most commonly cited CAM modalities included yoga (14.0%), evening primrose (13.1%), imagery/visualization (10.4%), meditation (7.7%) and music therapy (5.6%). Similarly, physicians rated meditation and guided imagery as highly or moderately effective forms of CAM. However, other CAM modalities rated as highly or moderately effective by physicians (biofeedback, chiropractic and acupuncture) were not commonly used by patients. It also notable that CAM modalities least recommended by physicians (such as aromatherapy, bioelectromagnetic therapies, and homeopathy) were rarely used by patients.

**Table 4:** Patients' ever use of CAM modalities for obstetric and/or gynecologic problems

**Modality**	**Ever Used for Obstetric and/or Gynecologic Problems (%)**
Yoga	14.0
Evening primrose	13.1
Imagery/visualization	10.4
Meditation	7.7
Music therapy	5.6
Soy	4.8
Chiropractic	4.8
Journaling	3.7
Acupuncture	3.3
Ginger	3.1
Black cohosh	2.9
Chinese herbs	2.9
Aromatherapy	2.7
Homeopathy	2.3
Vegetarian diet	2.5
Tai chi/Chi gong	1.9
Juicing diet	1.9
Ayurvedic remedies	1.7
Energy healing	1.7
Echinacea	1.5
Osteopathic	1.5
Vegan diet	1.3
Magnet therapy	1.0
Hypnosis	0.8
Biofeedback	0.8
Immune therapy	0.8
False unicorn root	0.6
Macrobiotic diet	0.2
Healing touch	0
Reflexology	0

The most commonly used dietary supplements were evening primrose and soy. The majority of patients reported using CAM interventions for pregnancy symptoms, menstrual, or menopausal symptoms. Other frequently reported reasons for CAM use included infertility, pelvic pain, and libido.

### Sources of CAM Information

Two hundred eighty seven patients responded to the question, "If you currently use or have used alternative therapies, how did you find out about them?" The most commonly cited source of CAM information was through family and friends (n = 104, 36.2%). Other less commonly cited sources of CAM information included the Internet (n = 48, 16.7%), a health care professional (n = 45, 15.7%), and books (n = 45, 15.7%). Most patients (63%) did not consult with a health care provider prior to starting the alternative therapy. The most commonly cited reason (43%) was that their health care provider never asked about their use of other therapies. Among the patients who did consult their healthcare provider prior to starting CAM therapy (29.2%), most patients noted that physicians' response was positive and that they encouraged continued use of CAM (58%).

## Discussion

In the U.S., CAM use is prevalent, particularly among women, where 39% have reported using CAM [11]. Our data is consistent with CAM use reported previously for women in the state of Michigan in 2001 (53.8%) [22]. The obstetrician gynecologist may play an integral role in incorporating CAM use with conventional medicine among this patient population. We therefore sought to determine general attitudes and approaches to CAM among obstetric and gynecology patients and physicians. Despite the fact that both physicians' and patients' attitudes toward CAM were generally positive in this study, physicians' and patients' responses were not identical. Surprisingly, we found that physicians appeared to have a more positive attitude towards CAM as compared to general obstetric/gynecology patients.

Differences between physician and patient attitudes towards CAM may be influenced by several factors. It is notable that among the patients surveyed, a significant portion of respondents had neutral responses to the general attitude questions (Table 1), or skipped the question altogether, possibly indicative of an ambivalent stance. If the neutral/skipped responses had not been included in the chi squared analysis, differences between physician and patient responses may not have been as pronounced. Physician and patient beliefs regarding different types of CAM may be influenced by personal experience. Furthermore, physicians and patients perception of the definition of CAM may vary. For example, the concept of "faith healing" may be difficult to distinguish from "spiritualism" or from "prayer" in general [23-25]. It has been reported that one of the largest subgroups of CAM users is educated, employed women [3,11]. Although we did not have demographic data available from the patients surveyed, differences in age, education level, and other demographic factors may contribute towards the differences seen between physician and patient attitudes towards CAM.

Although the overwhelming majority of physicians surveyed indicated that they referred patients for at least one CAM modality, we found that over 63% of obstetric/gynecologic patients surveyed that used CAM, initiated CAM therapy without consulting a physician. It is not surprising then that physicians' view of the most effective CAM therapies were incongruent with the therapies most used by patients. Physicians most commonly cited biofeedback, chiropractic, acupuncture, meditation and hypnosis/guided imagery as being highly/moderately effective. In contrast, the most commonly cited CAM modalities used by patients were yoga, evening primrose and music therapy. In addition, in contrast to the physician survey, the patient survey fragmented the herbal remedies surveyed into different plants. If all herbal remedies surveyed are combined, herbal remedies are the most common modality used by patients in this study. In general, although more than 50% of physicians endorsed the use of movement therapies, biofeedback, acupuncture, meditation, chiropractic, and hypnosis/guided imagery, these modalities were rarely used among patients (Tables 3 and 4).

The majority of patients who initiated CAM without consulting their healthcare provider prior to initiating a CAM therapy indicated that they did so because their physicians never asked them about their use of CAM. In contrast, 83% of physicians surveyed indicated that they routinely query their patients about CAM use. This discrepancy could be due to the fact that physicians only ask a portion of their patients about CAM use and not all patients. Due to a trend toward managed care and shorter office visits, physicians have limited time to spend with patients. Time constraints may render discussion and integration of CAM therapies into mainstream practice difficult. For example, there is some evidence that incorporating discussion of CAM may double consultation time [26]. However, without discussion of CAM therapies, a patient's medical record is incomplete and the possibility of medical risk cannot be addressed.

While some CAM therapies impose risks to patients, there are several CAM therapies which have shown benefit. For example, it has been demonstrated that the use of moxibustion can increase the rate of spontaneous version from breech to vertex in pregnant women at term [27,28]. CAM interventions such as Tai Chi, acupuncture, acupressure, yoga, and meditation have improved sleep parameters in a limited number of early clinical trials [29]. On the other hand, herbal remedies, considered to be both safe and effective by most consumers, may interact with conventional drugs, such as Coumadin [30]. An increasing number of CAM therapies have shown evidence based benefits, which is likely why the majority of patients indicated that physicians encouraged continued use of CAM.

It is important for obstetrician gynecologists to remember that many CAM therapies are still not subject to standardized manufacturing or regulation by the U.S. Food and Drug Administration. Thus, there can be extreme variation in each therapy and safety is still a prominent issue. Physicians must be responsible for discussing the safety of CAM modalities and how they may be incorporated with conventional medicine. In our study, 98.4% percent of physicians have endorsed/provided/or referred a patient for at least one CAM therapy. Healthcare networks do exist which aim to integrate both medical doctors and alternative medicine practitioners. Some of these networks provide access to credentialing information on CAM practitioners and offer a centralized medical record system which creates an avenue for both medical doctors and CAM practitioners to communicate, enhancing the care of the patient [31].

Our study has several limitations. First, the response rate for physicians was 41% and 32% for physicians and patients respectively, which may reflect self selection and lead to response bias. Questionnaires for this study were modified from previously published studies [32,33]. We did not obtain demographic data for patients which may have added important information regarding CAM use in this population. In addition, the physician questionnaires did not delineate effectiveness of CAM modalities "for what," nor whether a modality was being judged as complementary or alternative. Questionnaires were designed to be very basic and abbreviated in order to encourage response. In addition, all data was self-reported and therefore subject to recall bias. Our survey was further limited by sample size which required us to combine CAM modalities when determining factors that correlated with view of effectiveness and use of CAM in patients and physician practice. Small sample size leads to limited power to detect small differences. Finally, the prevalence of CAM use among patients and physicians is oftentimes reported as any use of CAM during the last year; in our study, we queried "ever use" of various CAM modalities. Comparisons between our study and previously published work must take this into consideration.

It is also important to note that our patient population was limited to one University-based outpatient clinic and the physician population was limited to the state of Michigan which may not be representative of other states. The results of this survey should be generalized in a cautious manner secondary to the limitations noted above. Additional studies are necessary to incorporate larger patient samples which would be more representative of the population in the United States for both patients and obstetrician gynecologists. In future studies, it would be important to examine objective measures of physicians' use of CAM modalities in contrast to self-report. Longitudinal studies are necessary to follow physicians' use of and attitude toward CAM as it becomes further integrated into medical education and more evidence based information is obtained.

Despite these limitations, it is clear that physicians must educate themselves in the field of complementary and alternative medicine in order to give accurate advice to patients to optimize their care. In 1998, incorporation of CAM training occurred in 64% of academic medical institutions [34]. Options for integrating CAM instruction at the postgraduate levels are more limited; at present there are only a few academic institutions in the U.S. with formal CAM education programs in place. The need for dialogue between physician and patients regarding CAM use is clear, as patients are increasingly seeking physicians who are well-informed in the realms of both conventional medicine and CAM.

## Competing interests

The authors declare that they have no competing interests.

## Authors' contributions

MLF and JRL designed the study, collected data, and drafted the manuscript. DAP and AS performed the statistical analysis and helped to draft the manuscript. All authors read and approved the final manuscript.

## Pre-publication history

The pre-publication history for this paper can be accessed here:


